# Maternal Dietary Supplementation with γ-Aminobutyric Acid Alleviated Oxidative Stress in Gestating Sows and Their Offspring by Regulating GABRP

**DOI:** 10.3390/ani12192539

**Published:** 2022-09-22

**Authors:** Xiaoyi Liu, Lili Jiang, Jiaman Pang, Yujun Wu, Yu Pi, Jianjun Zang, Junjun Wang, Dandan Han

**Affiliations:** State Key Laboratory of Animal Nutrition, College of Animal Science and Technology, China Agricultural University, Beijing 100193, China

**Keywords:** gamma-aminobutyric acid, gestating sows, newborn piglets, pTr-2 cell, oxidative stress

## Abstract

**Simple Summary:**

Sows usually suffer oxidative stress during gestation, and this limits the growth of fetuses via placenta. Gamma-aminobutyric acid (GABA) is a functional nonessential amino acid engaged in regulating the physiological status of animals. However, the effects of GABA on the oxidative homeostasis of sows and their offspring remain unclear. This study aims to investigate the effects of GABA on the oxidative stress of gestating sows, newborn piglets, as well as the H_2_O_2_-treated porcine trophectoderm cell line-2 (pTr-2 cells) and to subsequently provide guidance on the application of GABA in sows’ nutrition during late gestation. We found that GABA alleviated placental oxidative stress in gestating sows by increasing antioxidant-associated enzyme activity and by upregulating the gene expressions of gamma-aminobutyric acid receptor (*GABRP*) and nuclear factor-erythroid 2-related factor-2 (*Nrf2*) in pTr-2 cells, as well as enhanced the antioxidant capacity of sows and fetal piglets. This study provides the possibility for future application of GABA in maternal–fetal nutrition.

**Abstract:**

Sows usually suffer oxidative stress during gestation, and this limits the growth of fetuses via placenta. Gamma-aminobutyric acid (GABA) is a functional nonessential amino acid engaged in regulating the physiological status of animals. However, the effects of GABA on the oxidative homeostasis of sows and their offspring remain unclear. Eighteen late gestating sows (85 d) were divided into the CON and GABA groups and fed the basal diet and the GABA diet (200 mg/kg GABA), respectively, until farrowing. At parturition, the sows’ litter characteristics, the plasma antioxidant parameters of sows, and their offspring were evaluated. The results showed that GABA supplementation had no marked effect on the reproductive performance of sows (*p* > 0.10) but had a trend of reducing the amount of intrauterine growth restriction (IUGR) in piglets (0.05 < *p* < 0.10). At the same time, the addition of GABA elevated the plasma superoxide dismutase (SOD) level of sows and enhanced the glutathione peroxidase (GSH-Px) activity of newborn piglets (*p* < 0.05). Based on the H_2_O_2_-induced oxidative stress in pTr-2 cells, GABA elevated intracellular GSH-Px, SOD, catalase (CAT), and total antioxidant capacity (T-AOC, *p* < 0.01) and upregulated the gene expressions of *CAT*, gamma-aminobutyric acid receptor (*GABRP*), and nuclear factor-erythroid 2-related factor-2 (*Nrf2*) in H_2_O_2_-treated pTr-2 cells (*p* < 0.05). Taken together, GABA improved the antioxidant capacity of sows and alleviated the placental oxidative stress by upregulating the *GABRP* and *Nrf2* genes, which have the potential to promote oxidative homeostasis in newborn piglets.

## 1. Introduction

Gestational sows accelerate their catabolism to meet the needs of fetal growth and development, which causes a metabolic burden and further oxidative stress [1,2,3]. Especially in late gestation, sows are more susceptible to oxidative stress [4]. The placenta, a transitional organ exchanging nutrients between the mother and the fetus, plays a key role in maintaining the growth of the fetus throughout gestation [5]. Impaired placental function and insufficient transport efficiency will alter the maternal–fetal interface and ultimately lead to fetal growth retardation [3,6]. IUGR is defined as impaired growth and development of the mammalian conceptus (embryo/fetus and its related placental membranes) or its organs during gestation, and it is usually seen in mammals, including humans and swine [7]. It is commonly believed that impaired placental growth leads to IUGR in mammals [8]. Embryonic trophoblast cells are one of the components of the placenta that can infiltrate and fuse with the endometrium and are closely related to the colonization of early embryos in the uterus. The normal development of trophoblast cells is a prerequisite to ensuring the formation and function of placenta and the development of embryos [9,10]. Therefore, oxidation stress during gestation will impair sows, ultimately affecting the growth and development of fetuses via the placenta.

Gamma-aminobutyric acid (GABA) is a natural important functional nonessential amino acid that has several physiological functions, such as anti-inflammation, anti-apoptosis, and anti-oxidation [11,12,13,14]. Although GABA is an inhibitory neurotransmitter in the mammalian central nervous system, GABA signals also widely exist in peripheral tissues and organs, such as the ovaries, placenta, and uterus [15,16,17]. At the same time, early studies have shown that GABA can activate the GABA receptor π subunit (GABRP) to regulate the development of human chorionic trophoblast cells [18,19]. According to these results, we can find that GABA has the effects of regulating physiological status, but the effects of GABA on oxidative stress of placenta, gestating sows, and their newborn piglets have not been reported.

Therefore, this study aims to investigate the effects of GABA on the oxidative stress of gestating sows, newborn piglets, as well as the H_2_O_2_-treated pTr-2 cells and to subsequently provide guidance for the application of GABA in sows’ nutrition during late gestation.

## 2. Materials and Methods

### 2.1. Animals, Diets, and Experimental Design

The animal use protocols were reviewed and approved by the China Agricultural University Animal Care and Use Committee (AW70302202-1-2, Beijing, China). The animal experiment was conducted in FengNing Swine Research Unit of China Agricultural University (Academician Workstation in Chengdejiuyun Agricultural and Livestock Co., Ltd., Hebei, China). Sows were randomly assigned to 2 groups in a completely randomized design. Briefly, eighteen Landrace × Large White multiparous gestating sows (initial BW in CON and GABA, 216.75 ± 4.57 and 213.75 ± 4.59, respectively; parity, 2–6; average parity in CON and GABA, 4.75 ± 0.36 and 4.50 ± 0.46, respectively) on day 85 of gestation were chosen and randomly divided into the CON and GABA groups according to the principle of similar average body weight and parity (*n* = 9 per treatment). The CON diet and the GABA diet were given from day 85 of gestation until farrowing. The feed allowance was set as 3.0 kg/d and divided into two equal rations. All sows had free access to feed and water from day 85 of gestation until delivery. Sows were housed individually in the gestation house from days 85 to 109 of gestation and transferred to the farrowing house individually on day 110 of gestation.

The basal diet was formulated on the basis of the nutritional needs of gestating and lactating sows according to the NRC 2012, and the GABA diet was composed of the basal diet plus GABA (200 mg/kg GABA, provided by the Shandong Tianyi Technology Co., Ltd., Jinan, China). GABA was mixed into the basal diet, and the CON group was also supplemented with alanine as an isonitrogen control. The composition and nutrient level of the diet are shown in Table 1. The birth weight, litter size, live piglets, stillborns, and IUGR piglets were recorded.

### 2.2. Sample Collection

The sows naturally farrowed, and parturitions were not induced. Gestation duration was mostly about 114–116 d. On the day of farrowing, blood samples were collected (anticoagulant: sodium heparin) from 18 sows and 1 piglet each from 6 of 9 litters from each treatment, with 3 male and 3 female piglets (without access to colostrum); half of the piglets were male, and half of the piglets were female. The blood samples in the sows and piglets were collected from the ear vein and jugular vein, respectively. Additionally, plasma was obtained by centrifuging at 3500 rpm at 4 °C for 10 min and stored at −80 °C for further analysis.

### 2.3. Oxidative Stress Evaluation

Relative glutathione peroxidase (GSH-Px), superoxide dismutase (SOD), catalase (CAT), total antioxidant capacity (T-AOC), and malondialdehyde (MDA) concentrations in the plasma were measured using ELISA kits (Jiangsu Feiya Biotechnology Co., Ltd., Yancheng, China), following the manufacturer’s instructions. 

### 2.4. Cell Culture and Treatment

The porcine trophectoderm cell line-2 (pTr-2) was kindly provided by Prof. Zhenlong Wu (China Agricultural University, Beijing, China). pTr-2 cells were cultured in DME/F12 medium (Gibco, New York, NY, USA) containing 10% (*v*/*v*) fetal bovine serum (Gibco, New York, NY, USA) and 0.1% (*v*/*v*) insulin (5 mg/mL, Solarbio, Beijing, China) at 37 °C and 5% CO_2_. Cells were treated with GABA (0, 25, 50, 100, or 200 μmol/L) for 24 h prior to stimulation with 0.6 mmol/L H_2_O_2_ for 2 h. 

### 2.5. Cell Viability Assay

pTr-2 cells were cultured in 96-well plates. After treatment, the supernatant was replaced by 100 μL of fresh medium containing the 10% Cell Counting Kit-8 solution and the cells were incubated for 2 h at 37 °C. The absorbance of each well was detected at 450 nm using the microplate reader (SpectraMax M2, Molecular Devices, San Jose, CA, USA). 

### 2.6. RNA Isolation, cDNA Synthesis, and Real-Time Quantitative PCR (RT-qPCR)

Total RNA was extracted from the cells with the Trizol reagent (Invitrogen, Carlsbad, CA, USA) and reversed into cDNA by using the Prime Script™ RT Kit (Takara, Shiga, Japan). RT-qPCR was performed according to the SYBR Premix Ex Taq™ II instructions (Takara, Shiga, Japan) on a Light Cycler^®^ System (Roche, CA, USA). The primers were synthesized by Sangon Biotech Co., Ltd. (Shanghai, China). The primer sequences are shown in Table 2. Amplifications were performed in triplicate for each sample. β-actin was regarded as the housekeeping gene, and the relative expressions of target genes were calculated according to the 2^−ΔΔCt^ method. 

### 2.7. Statistical Analysis

All of the data were analyzed in GraphPad Prism (version 8, GraphPad Software, San Diego, California, USA), and the results were shown as the mean ± SEM (standard error of mean). The Kolmogorov–Smirnov test was first carried out to verify if the data were normally distributed. For the sow experiment, Student’s t-test was used for a two-group comparison, the treatment factor GABA was used in the analysis, and the dependent variable in the model was the reproductive performance index. For the p-Tr2 cells experiments, one-way ANOVA was used for multiple comparisons; treatment factors including H_2_O_2_ and GABA were used in the analysis; and the variables in the model included cell viability, antioxidant parameters, and gene expression index. For all analyses, *p* < 0.05 means significant difference.

## 3. Results

### 3.1. Effects of Maternal Supplementation with GABA during Late Gestation on Litter’s Characteristics of the Sows

The litter’s characteristics are shown in Table 3. Compared with the CON group, maternal supplementation with GABA had no effect on the total piglets, live piglets, and stillborns. There was no difference in total litter weight and average birth weight between the GABA group and the CON group. However, maternal supplementation with GABA had a tendency to reduce the number of IUGR piglets (0.05 < *p* < 0.10). 

### 3.2. Effects of Maternal Supplementation with GABA during Late Gestation on Plasma Oxidative Stress of Sows

The increased burden of maternal metabolism in order to meet the large demand of nutrients for fetuses during gestation leads to oxidative stress [1,2,3]. To define the oxidative stress status, the plasma biochemical parameters of the sows were detected (Table 4). Compared with the CON group, maternal supplementation with GABA had no effect on the plasma GSH-Px and CAT activity of sows. There was no difference in the plasma content of MDA between the GABA group and the CON group. However, maternal supplementation with GABA increased the plasma SOD activity of sows compared with the CON group (*p* < 0.05). 

### 3.3. Effects of Maternal Supplementation with GABA during Late Gestation on Plasma Oxidative Stress of Piglets

During gestation, maternal oxidative stress impairs placental function and transport efficiency, which leads to fetal growth retardation [3,6]. To define the oxidative stress status, the plasma biochemical parameters of piglets were detected (Table 4). Compared with the CON group, maternal supplementation with GABA had no effect on the plasma SOD and CAT activity of piglets. There was no difference in the plasma content of MDA between the GABA group and the CON group. However, maternal supplementation with GABA increased the plasma GSH-Px activity of the piglets (*p* < 0.05).

### 3.4. Effects of GABA on H_2_O_2_-Induced Cell Viability of pTr-2 Cells

**Figure 1:**
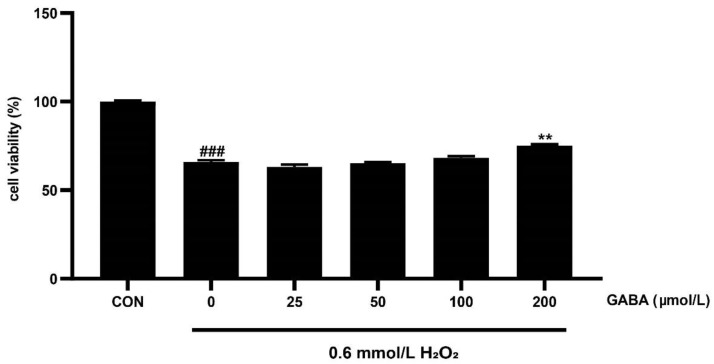
The effects of GABA on H_2_O_2_-induced cell viability of pTr-2 cells. CCK-8 method to detect cell viability. All data are presented as mean ± SEM and analyzed by one-way ANOVA. ### *p* < 0.001, compared with CON cells; ** *p* < 0.01, compared with H_2_O_2_-treated cells without GABA pretreatment. *n* = 6.

The abnormal development of embryonic trophoblast cells could affect the structure and function of placenta, and the transportation of nutrients, resulting in insufficient embryo development [9,10]. We then investigated the functional role of GABA on pTr-2 cells. As shown in Figure 1, compared with the CON group, H_2_O_2_ reduced the cell viability (*p* < 0.01). Compared with the H_2_O_2_ treatment, various concentrations of GABA (25, 50, and 100 μmol/L) pretreatment did not influence the cell viability, while the 200 μmol/L GABA pretreatment increased the cell viability (*p* < 0.01).

### 3.5. Effects of GABA on H_2_O_2_-Induced Antioxidant Parameters of pTr-2 Cells

**Figure 2:**
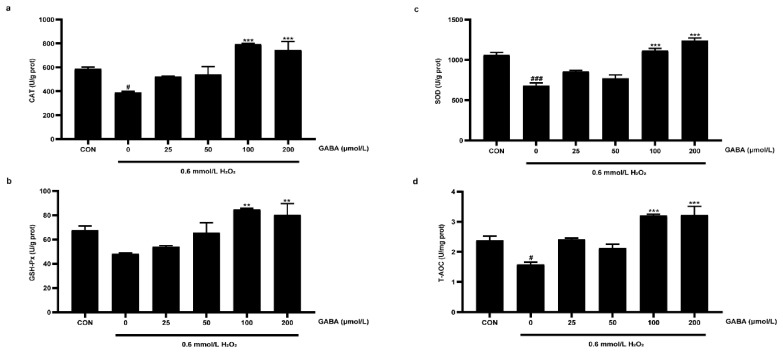
The effects of GABA on H_2_O_2_-induced antioxidant enzyme activity of pTr-2 cells. The enzyme activity of CAT (**a**), GSH-Px (**b**), SOD (**c**), and T-AOC (**d**). CAT, catalase; GSH-Px, glutathione peroxidase; SOD, superoxide dismutase; T-AOC, total antioxidant capacity. All data are presented as mean ± SEM and analyzed by one-way ANOVA. # *p* < 0.05, ### *p* < 0.001, compared with CON cells; ** *p* < 0.01, *** *p* < 0.001, compared with H_2_O_2_-treated cells without GABA pretreatment. *n* = 4.

To study the effect of GABA on oxidative stress of pTr-2 cells, the antioxidant enzyme activity was determined (Figure 2). Compared with the CON group, the H_2_O_2_ treatment decreased SOD (*p* < 0.01) and CAT (*p* < 0.05) activities. Compared with the H_2_O_2_ treatment, the 100 and 200 μmol/L GABA pretreatments increased the activity of GSH-Px, SOD, and CAT (*p* < 0.01). Furthermore, H_2_O_2_ induced a decrease in T-AOC (*p* < 0.05), while 100 and 200 μmol/L GABA pretreatment enhanced the T-AOC activity (*p* < 0.05). 

### 3.6. Effects of GABA on Gene Expression of CAT, SOD-1, Nrf2, and GABRP in H2O2-Induced pTr-2 Cells

**Figure 3:**
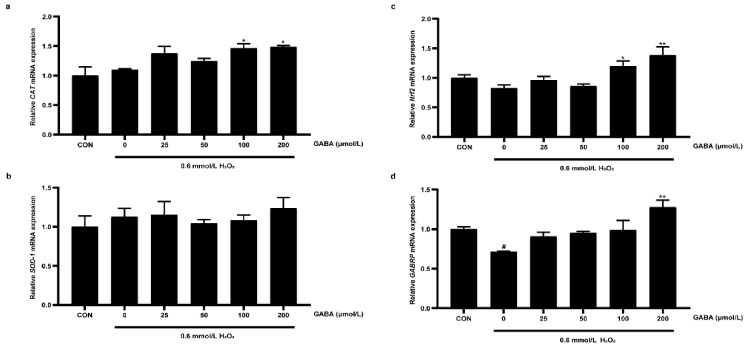
The effects of GABA on H_2_O_2_-induced gene expression of pTr-2 cells. The relative mRNA expression of CAT (**a**), SOD-1 (**b**), Nrf2 (**c**), and GABRP (**d**). CAT, catalase; SOD-1, Cu/Zn-superoxide dismutase; Nrf2, nuclear factor-erythroid 2-related factor-2; GABRP, GABAA-pi. All data are presented as mean ± SEM and analyzed by one-way ANOVA. # *p* < 0.05, compared with CON cells; * *p* < 0.05, ** *p* < 0.01, compared with H_2_O_2_-treated cells without GABA pretreatment. *n* = 4.

Nrf2 is a key factor related to oxidative stress, and GABA mainly exerts effects by binding to its receptor [14,21]. The mRNA expression levels of CAT, SOD-1, Nrf2, and GABRP of pTr-2 cells were determined (Figure 3). Compared with the H_2_O_2_ treatment, the 100 and 200 μmol/L GABA pretreatments increased the gene expression of CAT (*p* < 0.05). Additionally, the GABA pretreatment reversed the decrease in Nrf2 mRNA expression induced by H_2_O_2_ (*p* < 0.05). Compared with the CON group, H_2_O_2_ decreased the gene expression of GABRP (*p* < 0.05), which was recovered by the 200 μmol/L GABA pretreatment (*p* < 0.01).

## 4. Discussion

Increased burden of maternal metabolism leads to oxidation stress during gestation, which affects the growth and development of the fetus [22,23]. The placenta serves as the link between the mother and fetus to meet the needs of fetal growth and development [9]. The normal formation of placenta during gestation is closely related to the normal development of the fetus. As a major part of the placenta, abnormal proliferation and development of the embryonic trophoblast cells or oxidative damage of the placenta may lead to pathological phenomena such as IUGR and miscarriage [24,25]. As an important functional nonessential amino acid, GABA has been proven to have several physiological functions [11,12,13,14]. However, the effects of GABA on the oxidative stress of placenta, gestating sows, and their newborn piglets have not been reported. This experiment first carried out animal experiments to explore the effects of maternal supplementation with GABA on the oxidation status of gestating sows suffering from oxidative stress and their piglets. Secondly, an in vitro oxidative stress model induced by H_2_O_2_ on the pTr-2 cells was used to explore the potential mechanism of GABA on anti-oxidation. We hypothesized that GABA enhances the antioxidant capacity of sows and the placenta by regulating GABRP and Nrf2, thereby improving the antioxidant capacity of fetal piglets and, as a result, reducing the occurrence of IUGR.

Antioxidants play an important role in alleviating oxidative stress in sows. This study suggested that the supplementation of 200 mg/kg of GABA in late-gestating sows has no remarkable influence on the sow reproductive performance but has a trend of reducing the number of IUGR piglets. The results also showed that the addition of 200 mg/kg of GABA to the sow’s diet during late gestation significantly increased the SOD activity in the sow’s plasma by 16.21% and improved the activity of GSH-Px in the plasma of newborn piglets with compared with the control group. Overall, GABA supplementation improved the antioxidant capacity of sows and newborn piglets to some extent. However, the addition of 200 mg/kg of GABA did not completely improve the antioxidant enzyme system in the body, which may be related to the level of GABA supplementation. Therefore, it is possible that a higher dose of GABA supplementation may have a more positive effect on improving the antioxidant system of gestating sows and piglets, which needs to be further studied.

Recent studies have found that oxidative stress can cause damage to the antioxidant enzyme system of human chorionic trophoblast cells [26]. Research on GABA in livestock and poultry production mainly focuses on resisting heat stress and regulating appetite [27,28,29], but the effects of GABA on alleviating the oxidative stress of embryonic trophoblast cells are still unknown. Our results found that oxidative stress induced by H_2_O_2_ reduced the activity of SOD, CAT, and GSH-Px, while the 100 and 200 μmol/L GABA pretreatments can significantly increase the activity of these enzymes. However, these results seem to be somewhat different from the in vivo results above, in which maternal supplementation with GABA had no marked effect on the plasma GSH-Px and CAT activity of sows. We speculate that the possible reason is that the vitro experimental environment is relatively constant and controllable, while the actual environment in the housing environment of sows is relatively complex and uncontrollable, such as the temperature and humidity [30], environmental microbiota [31], etc. In addition, the level of oxidative stress induced by H_2_O_2_ may not be totally the same as the level of oxidative stress exposed by sows in an actual production environment, which might also be another reason for the discrepancy between them. Overall, the above results indicated that GABA pretreatment can improve and repair the antioxidant enzyme system in pTr-2 cells induced by H_2_O_2_.

Nrf2 is closely related to the antioxidant capacity of cells, which is an important regulator when cells are subjected to oxidative stress [21]. Nrf2 can activate a variety of antioxidant-related genes, thereby protecting cells from oxidative damage [32]. Studies have confirmed that the oxidative damage of mouse placenta caused by deoxynivalenol can reduce the expression of *Nrf2* in placental cells [33]. Our results found that oxidative damage reduced the expression level of the *Nrf2* gene in pTr-2 cells, while GABA pretreatment can significantly increase the gene expression of *Nrf2*. Therefore, GABA intervention to up-regulate the expression of the *Nrf2* gene may be an effective way to protect pTr-2 cells from oxidative damage. Although our mechanistic explanations are superficial, previous studies have revealed different mechanisms of GABA and Nrf2 in the anti-oxidant properties. GABA could alleviate oxidative stress induced by H_2_O_2_ and a high-fat diet [34,35], while Nrf2 could activate antioxidant-related genes, including *CAT* and *SOD-1* [36,37,38]. These will provide good insights for us to explore the underlying mechanisms of GABA in alleviating oxidative stress in gestating sows.

GABA mainly exerts effects by binding to GABRP, which played an important role during the process of embryo implantation and placenta formation [14,39,40]. Previous studies have confirmed that GABRP is widely distributed in various tissues of sows during late gestation [15], and GABRP expression was significantly increased during the embryo implantation in human and mice [39,40], which suggested that GABRP may be important for embryo development and mother–fetus communication. Our results found that oxidative stress significantly down-regulated the expression of the *GABRP* gene in pTr-2 cells, while GABA pretreatment can increase the expression of the *GABRP* gene, indicating that GABA may exert an anti-oxidative effect by combining with GABRP.

## 5. Conclusions

In conclusion, GABA alleviated placental oxidative stress in gestating sows by increasing antioxidant-associated enzyme activity and by upregulating the gene expressions of *Nrf2* and *GABRP*, consequently possibly alleviating the oxidative impairment of sows, thereby enhancing the antioxidant capacity of sows and newborn piglets. Our results provide new insights into the application of GABA in maternal–fetal nutrition.

## Figures and Tables

**Table 1:** Composition and nutrient levels of basal diet (%, as-fed basis).

Items	CON
Corn	60.00
Wheat bran	16.00
Soybean meal	19.00
Soybean oil	1.00
Premix ^1^	4.00
Nutrient levels ^2^	
DM	90.41
DE (MJ/kg)	3159
CP	15.97
EE	2.36
Calcium	0.83
Total phosphorus	0.47
L-Lysine	0.86
L-Methionine	0.23
L-Tryptophan	0.18
L-Threonine	0.59
NDF	18.15
ADF	5.09

^1^ Premix provided the following per kg of feed: vitamin A, 240–300 KIU; vitamin D3, 50–125 KIU; vitamin E, 500 IU; vitamin K3, 45 mg; vitamin B1, 50 mg; vitamin B2, 150 mg; vitamin B6, 100 mg; vitamin B12, 0.5 mg; pantothenic acid, 450 mg; niacin, 650 mg; folic acid, 80 mg; biotin, 10 mg; Fe, 2.4–18 g; Cu, 0.2–0. 8 g; I, 10–50 mg; Zn, 1.5–3.6 g; Se, 5–12.5 mg; Ca, 100–250 g; P, 20 g; NaCl, 60–220 g; choline chloride, 10 g; ^2^ DM, dry matter; DE, digestive energy; CP, crude protein; EE, ether extract; NDF, neutral detergent fiber; ADF, acid detergent fiber.

**Table 2:** Primer sequences for RT-qPCR.

Genes	Sequences 5′-3′	Tm, °C
*Nrf2*	F: GAAAGCCCAGTCTTCATTGC R: TTGGAACCGTGCTAGTCTCA	52
*SOD-1*	F: ACCTGGGCAATGTGACTG R: TCCAGCATTTCCCGTCT	52
*CAT*	F: AACTGTCCCTTCCGTGCTA R: CCTGGGTGACATTATCTTCG	52
*GABRP*	F: CGGATCAGCGGCTAGTGTT R: CGGTGACTTCGTGGAGGAA	55

**Table 3:** Effects of GABA supplementation during late gestation on litter characteristics for the sows.

Items	CON	GABA	*p* Value
Total piglets (*n*)	12.50 ± 1.30	12.63 ± 0.59	0.93
Live piglets (*n*)	11.75 ± 1.29	12.13 ± 0.44	0.78
Stillborn (*n*)	0.75 ± 0.31	0.50 ± 0.26	0.55
Total litter weight (live, kg)	16.06 ± 1.83	18.79 ± 1.06	0.22
Average birth weight (live, kg)	1.38 ± 0.07	1.57 ± 0.10	0.14
IUGR piglets ^a^ (*n*)	1.00 ±0.38	0.25 ±0.16	0.09

CON = control group fed basal diet, GABA = GABA group fed basal diet plus GABA. *n* = 9 per treatment. ^a^ Intrauterine growth retardation (IUGR) piglets = piglets with birth weight two standard deviations below the mean birth weight of the population [20]. Values are means ± SEM and analyzed by Student’s *t*-test.

**Table 4:** Effects of maternal supplementation with GABA during late gestation on plasma biochemical parameters of sows and piglets.

Items	CON	CON(CV)	GABA	GABA(CV)	*p* Value
Sows					
CAT (U/mL)	19.42 ± 1.91	0.2944	19.67 ± 1.50	0.2294	0.92
GSH-Px (U/mL)	696.70 ± 59.54	0.2564	582.70 ± 58.91	0.3033	0.19
SOD (U/mL)	62.32 ± 2.84	0.1368	72.42 ± 2.57	0.1064	0.02
MDA (nmol/L)	7.20 ± 0.38	0.1474	6.61 ±0.43	0.1828	0.32
Piglets					
CAT (U/mL)	19.42 ± 1.94	0.2450	21.30 ± 1.51	0.1737	0.46
GSH-Px (U/mL)	663.90 ± 21.85	0.0806	779.80 ± 44.00	0.1382	0.04
SOD (U/mL)	64.98 ± 3.56	0.1344	69.90 ± 3.98	0.1394	0.38
MDA (nmol/L)	7.56 ± 0.58	0.1882	7.48 ± 0.55	0.1788	0.93

CAT, catalase; GSH-Px, glutathione peroxidase; SOD, superoxide dismutase; MDA, malondialdehyde; CV, coefficient of variance. CON = control group fed the basal diet, GABA = group fed the basal diet plus GABA. Values are means ± SEM and analyzed by Student’s *t*-test, *n* = 9, sows; *n* = 6, piglets.

## Data Availability

The data presented in this study are available from the corresponding author upon request.
